# Ethical Risk Factors and Mechanisms in Artificial Intelligence Decision Making

**DOI:** 10.3390/bs12090343

**Published:** 2022-09-16

**Authors:** Hongjun Guan, Liye Dong, Aiwu Zhao

**Affiliations:** 1 School of Management Science and Engineering, Shandong University of Finance and Economics, Jinan 250000, China; 2 Institute of Marine Economy and Management, Shandong University of Finance and Economics, Jinan 250000, China

**Keywords:** artificial intelligence decision making, ethical risk, risk factors, mechanism of action

## Abstract

While artificial intelligence (AI) technology can enhance social wellbeing and progress, it also generates ethical decision-making dilemmas such as algorithmic discrimination, data bias, and unclear accountability. In this paper, we identify the ethical risk factors of AI decision making from the perspective of qualitative research, construct a risk-factor model of AI decision making ethical risks using rooting theory, and explore the mechanisms of interaction between risks through system dynamics, based on which risk management strategies are proposed. We find that technological uncertainty, incomplete data, and management errors are the main sources of ethical risks in AI decision making and that the intervention of risk governance elements can effectively block the social risks arising from algorithmic, technological, and data risks. Accordingly, we propose strategies for the governance of ethical risks in AI decision making from the perspectives of management, research, and development.

## 1. Introduction

Artificial intelligence was first proposed by McCarthy in 1956 to describe the intelligent behavior of man-made objects. Today, AI is widely used in all walks of life, such as in face and fingerprint recognition and VR interactions, and has greatly enriched our daily lives and improved our efficiency. With the development of AI, intelligent decisions based on big data have also emerged, with the most well-known example being Google’s development of the robot AlphaGo, which defeated the top professional human Go player to achieve the ultimate victory. In contrast to traditional decision-making processes based on human experience, emotional states, and “limited rationality”, AI decisions are based on machine learning algorithms and underlying data to make judgments regarding how things are developing. In modern life, AI plays an increasingly important role in helping humans to make decisions and is seen as a process that can enhance the efficiency of human decision making [1]. Much of the information, advertising, sound, and images that people obtain from their smartphones or personal computers originate from AI search algorithms and intelligent choices and recommendations based on public browsing behavior; even credit assessment tools are based on intelligent decisions made by artificial intelligence through big data and cloud computing.

The ethical risks of AI decision making comprise ethical and moral issues related to human beings and society that arise from errors caused by data or algorithms, and the negative effects of these risks must be addressed in the development of artificial intelligence. Some examples of AI decision making ethical risks include the choice between the lives of pedestrians and drivers in the event of danger, infringement of the privacy rights of people involved in “human flesh searching” based on big data technology, and incorrect decisions made by “intelligent courts” that lack human feelings. AI often struggles to cope with complex decision-making scenarios because tacit knowledge such as customs, emotions, and beliefs is difficult to fully digitize and structure. At the same time, the question of whether future intelligent decision making in the era of strong AI will surpass or even replace human choices is the “moral dilemma” of ethical risk. It is not yet certain whether AI will take away human control and bring unpredictable social risks to humans, and these issues are increasingly raising concerns about AI decision making.

To regulate the direction of AI, in 2016, the US established a new National Science and Technology Council (NSTC) Subcommittee on Machine Learning and Artificial Intelligence and introduced the National Strategic Plan for AI Research and Development, which includes understanding and addressing the ethical, legal, and social implications of AI as one of its seven strategies [2]. In 2020, the European Commission officially launched in Brussels the White Paper on Artificial Intelligence, A European Pathway to Excellence and Trust, stating that the development of AI should be human-centered, sustainable, and under ethical control, respecting the fundamental rights of people and avoiding the problem of risks associated with AI decisions [3]. In 2021, the European Commission also published a proposal for an Artificial Intelligence Act, which proposes to address the risks of AI, develop a unified and trusted EU AI market, and protect the fundamental rights of EU citizens [4]. As early as 2007, Japan and South Korea had formulated relevant documents for robots, proposing that machines should be controlled by people, etc. [5]. In addition, the UK and Japan have set up ethics committees and data ethics centers focusing on AI, gradually bringing the ethical issues of AI into focus [6].

In 2017, in the Development Plan for a New Generation of Artificial Intelligence, China’s State Council observed that AI is in the process of rapid development, and that strict attention should be paid to its risk challenges to ensure its safe and healthy development [7]. In 2018, General Secretary Xi Jinping, while chairing a collective study on the current status and trends of AI development held by the Political Bureau of the CPC Central Committee, stressed that the study and prevention of potential risks in AI development are crucial to the healthy development of AI. In 2019, China’s new-generation AI development plan established a new-generation AI governance professional committee, which is fully responsible for AI, including ethical code research and normative governance work. The above-mentioned documents regarding AI and various committees indicate that AI decision making has received widespread attention worldwide, and the study of the ethical risks posed by AI is crucial to the development of humanity and AI technology.

In this paper, we explore the ethical risks and dimensions of AI decision making and dissect the mechanisms of action between the risks through rooted theory and system dynamics. The purpose of this paper is to provide references for the scientific prevention, precise response, and timely resolution of the ethical risks of AI decision making to ensure the healthy and sustainable development of AI.

The remainder of this paper is organized as follows. In Section 2 the relevant literature is reviewed; in Section 3 the ethical risks of AI decision making are identified and analyzed using rooting theory; in Section 4, the mechanisms of action of the ethical risks of AI decision making are analyzed using system dynamics and simulation experiments are conducted; and Section 5 presents the discussion and conclusions of this paper.

## 2. Literature Reviews

Ethics, as a moral constraint and norm, is a standard for evaluating the relationship between human beings and nature, but there is no uniform text or theoretical system in relation to it [8]. The ethics of AI guides the development of AI technology without conflicting with human interests, and is a guideline for technological development and accepted ethical standards from which to achieve co-development between intelligent technology, human beings, and nature [9]. With the development of technology, academics are gradually paying more attention to the risks posed by technology. Among them, the issue of the ethical risks associated with AI decision making is an important issue that has received the most attention from scholars. The earliest ethical research on AI decision making began with robots [10], which led scholars to worry about whether machine thinking would surpass or replace human thinking in decision making, and to consider major ethical risks such as human dignity and human existential crises. The lack of human emotions and the inability of robots to make complex decisions including the recognition of emotions, coupled with the inadequacy of laws and regulations in the area of ethics, will inevitably lead to “robots killing people”.

### 2.1. Research on the Ethical Risks of Artificial Intelligence Decision Making

Artificial intelligence decision making is based on limited data, programs, relevant algorithms, and other input conditions to develop the best possible strategy. However, technology itself comes with uncertainty, and coupled with the incomplete nature of the data, decisions that lack human emotions within them are subject to decision errors and may also largely alter even human decisions, resulting in ethical risks such as privacy breaches, risk to human life, and undermining social justice; these uncertainties are an important source of ethical risks. The study of the ethical risks of artificial intelligence decision making involves clarifying the ethical risks caused by the uncertainty of technology and the uncertainty of human complex emotional decision making, to effectively prevent and protect against these risks, and to enable intelligent decision making to develop in a strong direction. The sources of ethical risks in AI decision making include two major causes of risk: technological uncertainty and human limited rationality [11]. From a technological perspective, technological loss of control, misuse, and abuse of technology are the greatest sources of technological risk [12]. Specifically, intelligent algorithms, program design, and other technologies that exist in the whole process of AI decision making are specific sources of ethical risks [13]. From the perspective of human limited rationality, since the programming and data importation samples in intelligent decision making involve human decisions, humans are the main source of risk creation [14], and the ethical risks under AI decision making originate from the complex interactions among technology, humans, society, and nature.

### 2.2. Research on the Ethical Risk Governance of Artificial Intelligence Decision Making

In response to the ethical risks that may be posed by AI, many scholars have proposed risk governance, mainly through top-down governance and bottom-up governance measures. The top-down approach involves developing a framework with ethical and moral awareness, and ethical rules so that robots can be bound to make decisions and act within this framework. Examples include Amoff’s moral calculus [15], the three laws of robotics [16], Kant’s categorical imperative [17], or general moral philosophical content. In terms governance measures, risks in the decision-making process can be prevented through the development of a list of principles for new technologies [18], corresponding ethical risk governance framework guidelines [19], and governance systems [20]. However, all ethics and rules have their imperfections, coupled with the fact that human emotions are complex and influenced by a variety of values, social principles, etc., which cannot be generalized by mere rules. This makes it very difficult to develop intelligent decision-making systems based on a top-down approach. A bottom-up governance approach involves a machine building a system of ethical decisions close to human thinking patterns by continuously simulating the behavior and emotions of the person, similar to machine learning. The most famous example is autonomous driving technology; however, inaccurate knowledge of the rules by humans themselves can create bad habits in machines, which can lead to risks and even difficulties in decision making. Neither top-down nor bottom-up approaches to governance can make machines think like humans and have ethical awareness, either on a technical or a moral level. Some studies have shown that people are not opposed to the implementation of new technologies and that the main reason for people’s fear of AI decision making is based on a distrust of government [21], so it is particularly important to strengthen the potential ethical review and legal implications of the AI decision-making process [22] and to govern the ethical risks of AI decision making [23].

In general, the development of AI has become increasingly mature and its intelligent decision making has been applied to many aspects of human life [24], medicine [25], ecology [26], and social management [27]. However, as a technology, the intelligent decision making of AI inevitably poses corresponding ethical risks, and fewer studies have been able to summarize the risks and risk formation mechanisms of AI ethical decision making and investigate the relationships between the risks. In this study, we use a qualitative research method of rooted theory to identify and organize the risk factors of AI ethical decision making, including risk sources and risk consequences. We construct a conceptual model and a feedback model of risk factors through system dynamics to explore the formation mechanism of AI ethical risks, and analyze the causes of risks from multiple perspectives and in an all-round way, in order to provide effective assistance to ethical decision making and reduce the negative effects of AI ethics.

## 3. Identification of Ethical Risk Factors for AI Decision Making Based on Rooting Theory

### 3.1. Research Methods

**Figure 1:**
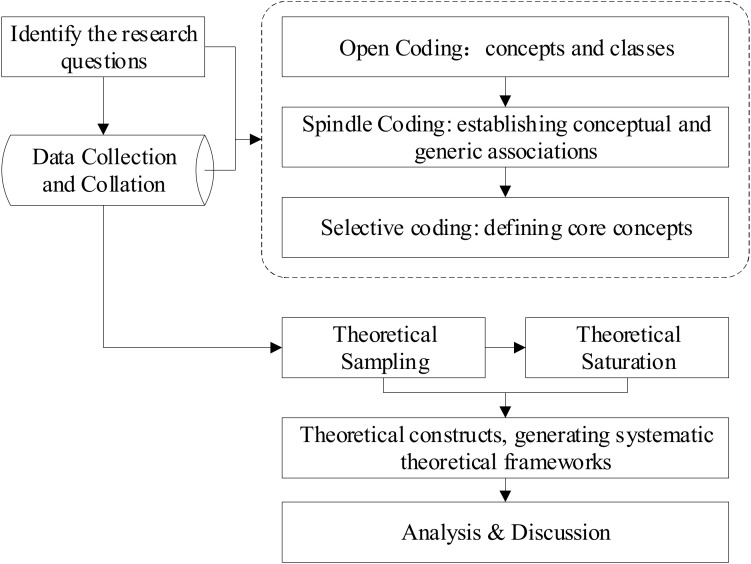
Process of rooting theory.

Qualitative research involves the human- and action-based study of social phenomena, and its common approach is evolutionary reasoning (Catherine, M., 2019, P4–5) [28]. Rooting theory is a common method used in qualitative research. Rooting theory is a fact-based theory of the induction and conceptualization of unstructured data based on data collection and interviews (Juliet, M.C., 2015, P48–49) [29]. It is a bottom-up simulation research process, and the resulting theory needs to be standardized in a process of continuous development and refinement. In this regard, rooting theory is divided into seven steps, as shown in Figure 1.

The seven steps are as follows: defining the research question → data collection and collation → open coding → spindle coding → selective coding → theory saturation testing → theory construction. Data collection and level-by-level coding are the two most important steps in rooting theory. Through the three-level coding process, complex data can be generalized and a complete and standardized theoretical model can be constructed.

### 3.2. Data Collection and Collation

Since the introduction of the concept of AI, there has been a proliferation of research on the subject. As a qualitative research method, rooting theory requires abundant data to support it. In this study, we followed the principle of “everything is data” and returned to the original literature. We used official websites, authoritative news media websites, Baidu, Zhihu, the China Knowledge Network, and relevant literature-reading websites in Chinese, as well as Google, Yahoo, Twitter, and other websites, to browse and collect various information related to the research topic and to obtain secondary data. The transcripts obtained include not only literature, but also reports and opinions related to AI ethics.

In terms of Chinese literature selection, the main focus was on the China National Knowledge Infrastructure. We used CSSCI (Chinese Social Sciences Citation Index) and CSCD (Chinese Science Citation Database) as the screening criteria. A total of 84 articles were obtained with the theme of “ethical risk of artificial intelligence”, and one article was obtained with the theme of “ethical risk of artificial intelligence decision making”. In addition, the keywords “ethical risks of AI decision making” were used in Baidu’s engine to filter out more reports from People’s Daily, Guangming Daily, etc. The research on the ethical risks of AI decision making is relatively limited and needed to be extracted from articles on the ethical risks of AI. In the process of English literature selection, Elsevier’s full-text journal database with “ethical risks of AI decision making” as the theme returned 587 articles in 2022, which also shows that other countries pay more attention to AI decision making and risks. However, Flynn [30] found that the number of articles on rooted theory was between 4 and 49; therefore, Flynn believed that a sample size of around 20 could guarantee the rationality of the theory.

**Figure 2:**
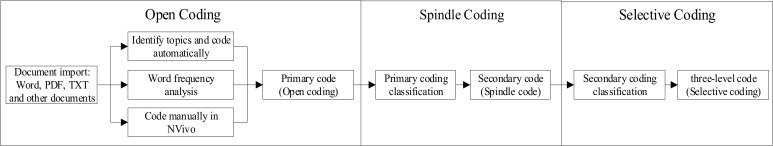
Three-level coding process in NVivo.

In this paper, NVivo is employed to collate the screened literature. NVivo is a powerful qualitative analysis software package that can import different types of data and collate these data. Two-thirds of the text data were randomly selected and imported into NVivo for deep data mining and collation. As the content regarding the ethical risks of AI decision making needed to be analyzed through the content of the article, we used the word frequency analysis and manual coding functions in NVivo to sort through the literature while performing manual coding to form the initial concept, after which the coding was compiled by the coding classification method for spindle coding and selective coding. The detailed process is shown in Figure 2. In addition, the remaining third of the literature was used for the theoretical saturation test.

### 3.3. Research Process

#### 3.3.1. Open Coding

Open coding is essentially the process of organizing and summarizing large sections of collected text into definitions in the form of concepts. Open coding consists of three steps. The first step is tagging, where textual statements are labeled. The second step is conceptualization, in which the labeled concepts are further analyzed, compressed, and simplified, and keywords are extracted to form a preliminary concept. The third step is scoping, in which the concepts are refined at a deeper level and further condensed into concepts. For example, the initial concept of “human-caused discrimination” is based on the original record entry “the introduction of discrimination or bias into the decision-making process by an algorithm for human reasons”. Due to space constraints, the original statements are not presented in this paper. In this study, the statements were annotated in NVivo and codes with the same or similar semantic meanings were combined, through discussion and analysis, to form 126 initial concepts. Based on the analysis and expansion of the meanings of each initial concept in the research context, the initial concepts were combined, resulting in 22 initial categories, as shown in Table 1.

#### 3.3.2. Spindle Coding

Spindle coding is a process that further categorizes and analyses initial categories based on the open coding results. Spindle code is used to discover potential logical relationships between the categories. By regrouping the information and mining the logical order and relationships between the 22 initial categories presented in Table 1, we considered the contextual characteristics of the study and categorized the initial categories. Finally, we obtained seven categories: algorithmic risk, data risk, technology risk, social risk, management risk, decision risk, and risk management, as shown in Table 2.

#### 3.3.3. Selective Coding and Theoretical Models

**Figure 3:**
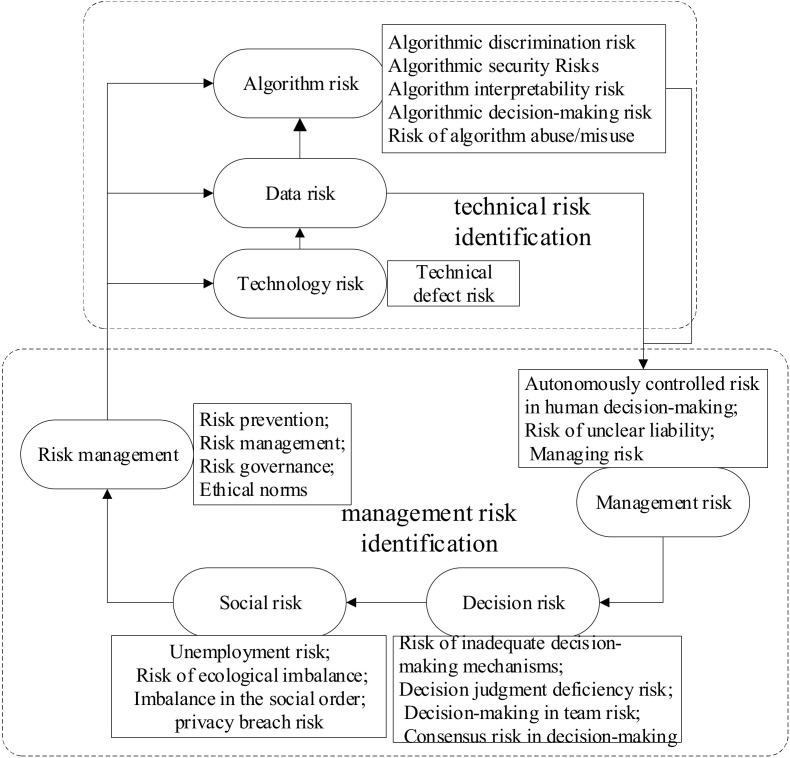
Conceptual model of ethical risk factors for AI decision making.

**Figure 4:**
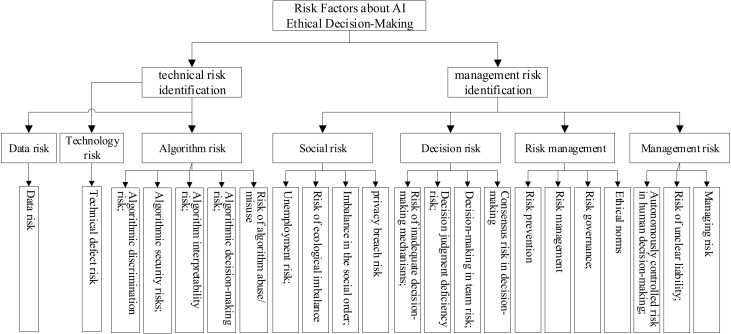
Structural model of the dimensions of ethical risk factors regarding AI decision making.

Selective coding refers to the distillation of the core categories from the main categories. The main categories are highly condensed through the core categories and linked to form a complete storyline, which leads to a theoretical model. In this paper, we obtained 22 categories and seven main categories. Finally, we obtained two core categories: technology risk identification and management risk identification. Technology risk identification includes algorithm risk, data risk, and technology risk, while management risk identification includes managing risk, decision risk, and social risk. In addition, both management risk and technical risk can be refined through risk management to reduce their occurrence, as shown in Figure 3. The ethical risks of AI decision making mainly include the existing risks in the technology itself and management risks. On the one hand, the development of technology is inherently uncertain, and the development of AI is at the forefront of technological development. There are also bound to be unknown ethical risks. Therefore, making the algorithms and technology transparent will be more helpful for decision making. On the other hand, the ethical risk of technology is human. The misuse or abuse of technology will cause ethical and social problems directly, so it is equally important to strengthen risk management. A conceptual model of the ethical risk factors in AI decision-making process is shown in Figure 3. The model of the dimensional structure of the ethical risk factors in AI decision making is shown in Figure 4.

#### 3.3.4. Theoretical Saturation

Theoretical saturation refers to the point at which no new concepts or categories can be generalized beyond those already collected, at which point the data collection and collation process can be stopped. In this study, it was found that the concepts obtained could be fully generalized to the resulting categories by coding, summarizing, and organizing the remaining third of the textual data in the same way. There were few relationships found between concepts and categories, indicating that the model was saturated.

## 4. Mechanisms for Ethical Risks in Artificial Intelligence Decision Making Based on System Dynamics

Ethical risk decision making in AI is a complex system with a large number of risk factors, and there are complex relationships and pathways of influence between factors. System dynamics is uniquely suited to the study of complex non-linear systems; it is used to qualitatively and quantitatively dissect the complex relationships and mechanisms of action between factors [35]. A causal analysis of system dynamics, based on the system structure, can treat the system as a causal feedback mechanism with multiple information, revealing the causal relationships, interactions, and dynamic changes of each influencing factor within the system. System dynamics is therefore an important tool with which to analyze the relationships between the factors and causal paths of action in complex systems.

### 4.1. Causal Construction

When using system dynamics for modeling and simulation, it is first necessary to identify the key variables in a complex system before the causality and flow diagrams of the AI ethical decision risk system can be plotted. In this study, the 26 variables were plotted into two causality diagrams based on the results of rooting theory and the influence relationship diagram, which indicate the change in ethical risk causes in the ungoverned state and the trend of risk change after risk governance.

#### 4.1.1. Risk Subsystem Causality Analysis

**Figure 5:**
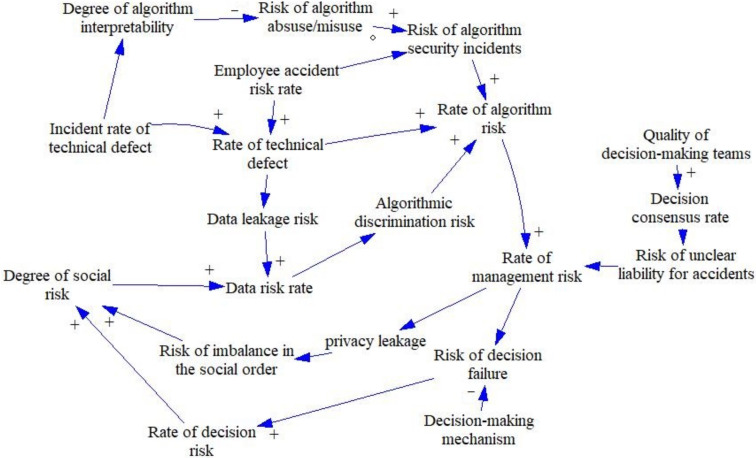
Risk subsystem causality diagram. Loop 1: degree of social risk (DSR) → data risk rate (DRR) → algorithmic discrimination risk (ADR) → rate of algorithm risk (RAR) → rate of management risk (RMR) → risk of decision failure (RDF) → rate of decision risk (RDR) → degree of social risk (DSR); Loop 2: DSR → DRR → ADR → RAR → RAR → privacy leakage (PL) → risk of imbalance in the social order (RISO) → DSR.

The system of ethical risk in artificial intelligence decision making involves the risk faced within the system without risk management, including the sources of risk and the consequences of risk. This risk system comprises technical risk, algorithmic risk, data risk, management risk, decision risk, and ultimately social risks of varying degrees of consequence, as shown in Figure 5. Two main loop systems exist for the AI decision ethics risk system: Loops 1 and 2.

#### 4.1.2. Risk Management Subsystem Causality Analysis

**Figure 6:**
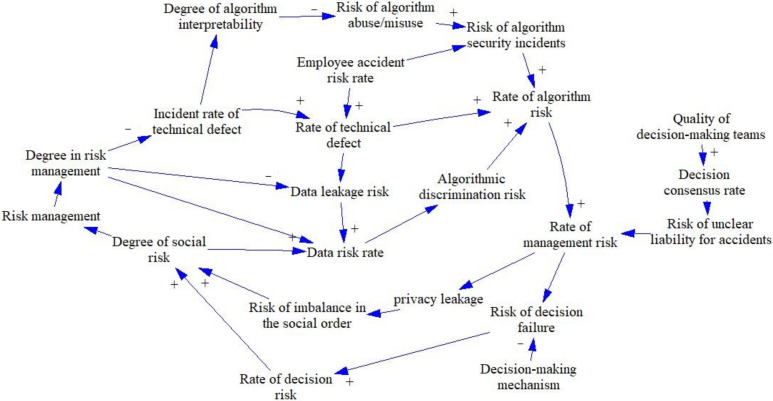
Risk management system causality diagram. Loop 3: degree in risk management (DRM) → DSR → ADR → RAR → RAR → data leakage risk (RDF) → RISO (RDF) → DSR → risk management (RM) → DRM; Loop 4: DRM → incident rate of technical defect (IRTD) → rate of technical defect (RTD) (→ data leakage risk (DLR) → DSR → ADR) → RAR → RAR → RDF (DLR) → RDF (RISO) → DSR → RM → DRM; Loop 5: DRM → DLR → DSR → ADR → RAR → RAR → RDF (DLR) → RDF (RISO) → DSR → RM → DRM; Loop 6: DRM → IRTD → degree of algorithm interpretability (DAI) → risk of algorithm abuse/misuse (RA A/M) → risk of algorithm security incidents (RASI) → RAR → RAR → RDF → RDF → RM → DRM. Note: “Underline” indicates the simultaneous alternative path.

The AI decision making ethical risk management system is based on a risk subsystem with risk management content, including risk governance, ethical norms, management systems, and preventive measures to compare the effectiveness of risk governance, as shown in Figure 6. There are eight circuits of the artificial intelligence decision making ethical risk management system, which are Loops 3–6.

### 4.2. System Flow Diagram

**Figure 7:**
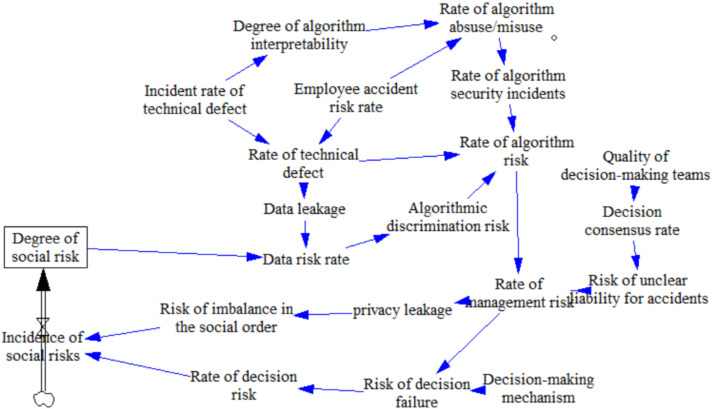
Risk subsystem flow diagram.

**Figure 8:**
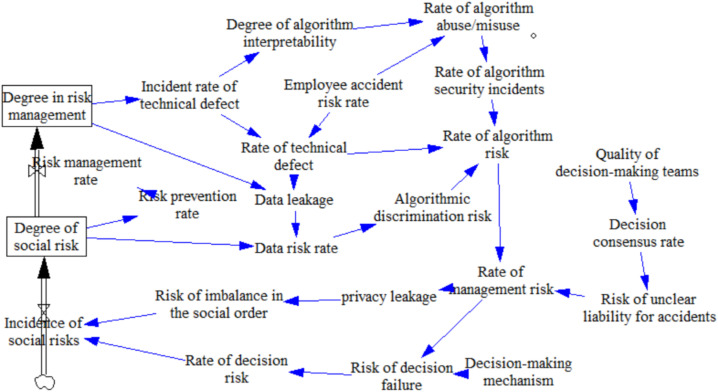
Risk management system flow diagram.

Causality diagrams and system feedback loops can reflect the basic institutions of a system dynamics model. They are qualitative analyses of the system model, but cannot indicate the nature of the variables in the system and the quantitative relationships between them. We used system flow diagrams to further analyze and explore the relationships between the effects of risk; to investigate the dynamic relationships between the nature, structure, function, and behavior of variables; and to create system flow diagrams to provide a basis for the establishment of model equations. We divided the AI decision making ethical risk system into a risk subsystem and risk management subsystem based on the causality diagram and system feedback loop, and used vensim PLE to draw the system flow diagram, as shown in Figure 7 and Figure 8.

### 4.3. Model Assumptions and Equations

In this study, according to the conceptual model of ethical risk in AI decision making, the variables were divided into level variables, rate variables, auxiliary variables, and constants. As this study focused on the evolutionary trend of risk and the state effect of risk under the condition of governance, the relevant data are simulated values. The variables were assigned data values based on the degree to which risk is described in relevant literature such as Zhang Tao [34], Lo Piano [36], the Artificial Intelligence Development Report (2018–2019), and the Ethical and Moral Standards for Artificial Intelligence introduced by the Defense Innovation Board under the US Department of Defense. It was assumed that the decision-making mechanism and team quality of the risk management organization did not change over six months and these were set as constants. The variables and key relationships are shown in Table 3.

The AI decision ethical risk management variables and the equations were based on Table 3 with the addition of the risk management module; most of the variables were the same, and the differences are detailed in Table 4.

### 4.4. Simulation and Testing

**Figure 9:**
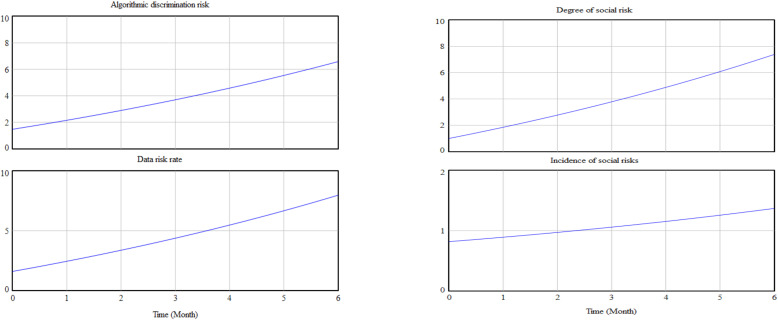
Pre-governance risk development.

**Figure 10:**
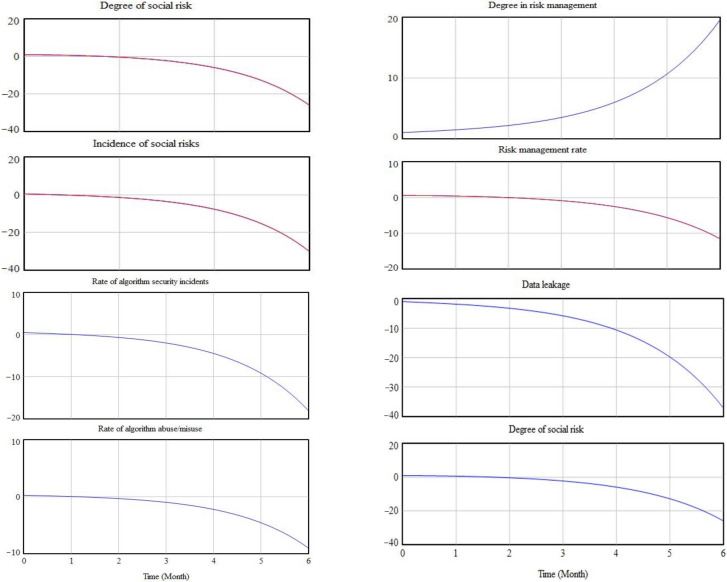
Risk development after governance.

Due to the lack of empirical research data on the ethics of ethical risk in AI decision making, it is not possible to compare experimental data with actual data. Therefore, we obtained more realistic results by iteratively adjusting the formulae and sensitivity tests, respectively, for the development of risk before governance and after governance. We used the vensim PLE simulation program to carry out simulation operations, taking initial time = 0, final time = 6, timestep = 0.125, and unit of time = month. We adjusted the parameter values of the artificial intelligence decision making ethical risk variables to obtain the changes in the risk subsystem and the governance subsystem. For example, Figure 9 shows the level of risk development before governance and Figure 10 shows the level of risk after governance.

The simulation results show that, over time, without the intervention of governance, algorithmic risk and data risk are in an uncontrollable state, resulting in a gradually increasing and uncontrollable rate of social risk. However, after the inclusion of the governance condition, although there was not a greater effect in the early stages (probably due to the low priority given to governance measures), in the later stages, as the degree of risk governance increased, both algorithmic risk and data risk rate showed a significant decrease. Thus, the degree of social risk also decreased, and the problem of ethical risks associated with AI decision making was better controlled.

## 5. Conclusions

While we enjoy the convenience brought by AI, we also need to avoid the ethical risks it may generate as far as possible. In this paper, we identified and organized the ethical risk factors of AI decision making using a rooted theory approach, and constructed a conceptual model of risk factors. A feedback model of risk factors was also constructed through system dynamics to explore the formation mechanism of the ethical risks of AI. We analyzed the causes of risk from multiple perspectives and in all aspects, to provide effective assistance in respect of ethical decision making, reduce the negative ethical effects of AI, and guarantee the healthy, long-term, and responsible development of AI, thereby promoting the level of governance of national science and technology development. The main findings and insights of this paper are as follows.

### 5.1. Ethical Risk Factors for Artificial Intelligence Decision Making

Based on the rooting theory, we obtained two core categories: “technical risk identification” and “management risk identification”. Technical risk identification includes algorithm risk, data risk, and technology risk, which occupies 36.5% of the first-level nodes. Management risk identification includes both management risks and risk management. The management risks include management risks, decision risks, and social risks caused by the three categories, occupying 39.6% of the first-level nodes, and risk management occupying 23.9% of the first-level nodes. Overall, it seems that technology and management risks in AI decision ethical risks have equal status and are two aspects that need to be focused on. The AI decision ethical risk factor dimensional structure model summarizes AI decision ethical risk factors and provides different dimensions for ethical decision making and evaluation in the future. In addition, the role of risk management is to reduce the occurrence of the risk, propose measures and solutions to reduce the occurrence of the risk, which will help prevent the occurrence of risk when making decisions, and enable AI to develop in a more healthy direction.

### 5.2. Ethical Risks of Artificial Intelligence Decision Making and Mechanisms of Governance

Based on the identification of ethical risk factors for AI decision making, the relationships and pathways between the risk factors were explored through the use of system dynamics. Vensim software was used to simulate the ethical risk model of AI decision making. From the causal loop perspective, on the one hand, the main factors that cause ethical risk in AI decision making are data risk and technology risk. The uncertainty of technology and the incompleteness and inadequacy of data can cause bias in decision making, which can lead to more serious ethical problems in technology. In addition, management failures can lead to serious social risks, such as unemployment. On the other hand, by adding risk management elements to the risk feedback model, the algorithm, technology, and data risk rate can be reduced significantly, thus effectively reducing the incidence of social risks.

### 5.3. Recommendations for the Governance of Ethical Risks in AI Decision Making

According to the factors and mechanisms of risk, it can be governed in terms of the management norms, R&D norms, and usage norms of risks. In terms of management norms, organizations related to AI technology development and application should strengthen risk identification and assessment in the process of technology promotion, promote agile governance, do a good job of prior control, and strengthen risk prevention. In terms of R&D norms, researchers should strengthen their sense of self-discipline, improve data quality, and guarantee safe and reliable data; algorithms should enhance security and transparency, and avoid biased discrimination of algorithms and data. In terms of usage norms, quality control should be strengthened, user rights should be safeguarded, and emergency protection should be enhanced, while the misuse or abuse technology should be avoided.

### 5.4. Contributions of this Research

In this study, we use a qualitative research method of rooted theory to identify and organize the risk factors of AI ethical decision making, including risk sources, risk consequences, and risk governance conditions, and we constructed a conceptual model of risk factors. We also constructed a feedback model of risk factors through system dynamics to explore the formation mechanism of AI ethical risks, and analyzed the causes of risks from multiple perspectives and in an all-round way. We find that technological uncertainty, incomplete data, and management errors are the main sources of ethical risks in AI decision making and that the intervention of risk governance elements can effectively block the social risks arising from algorithmic, technological, and data risks. Accordingly, we propose strategies for the governance of ethical risks in AI decision making from the perspectives of management, research, and development, with a view to providing effective assistance for ethical decision making and reducing the negative ethical effects of AI.

## 6. Limitations

There are some shortcomings in this paper. First, most of the previous studies on the ethics of science and technology have focused on major ethical issues in the field of life sciences, such as cloning, and less on the ethical risks in the field of artificial intelligence, and the collection of rooted data in this paper may not be sufficient. In addition, the rooted data sources in this paper are mostly texts such as literature and websites, which means that we may only identify the known risks of AI without further predicting and summarizing the unknown risks of AI. Therefore, the research on related issues in this paper needs to be further deepened. Finally, this paper constructs a system dynamics model based on qualitative analysis and constructs model relations based on previous scholars’ research and reports, and the simulation results lack the comparison and verification of realistic scenarios. There are various unpredictable risks and challenges in the development of AI, which need our attention in future research, and various research methods have yet to be tried, for example, collecting and storing data related to AI ethics, combining various methods for AI ethics research, and eventually building a general framework for AI ethics research.

## Figures and Tables

**Table 1:** Examples of open coding and scoping.

No	Initial Scope	Initial Concept
1	Algorithmic discrimination risk	Human-caused discrimination, data-driven discrimination, discrimination caused by machine self-learning, discriminatory algorithm design, non-discriminatory algorithm design, data bias, prejudice, discrimination, user equality
2	Algorithmic security risks	Algorithm vulnerabilities, malicious exploitation, algorithm design, training, algorithm opacity, algorithm uncontrollability, algorithm unreliability
3	Algorithm interpretability risk	Informed human interest and subjectivity, algorithmic transparency, algorithmic verifiability
4	Algorithmic decision-making risk	Algorithm prediction and incorrect decision making, unpredictability of algorithm results, algorithm termination mechanisms
5	Risk of algorithm abuse/misuse	Algorithm abuse, algorithm misuse, code insecurity, technical malpractice/misuse, over-reliance on algorithms
6	Technical defect risk	Limited technical competence, inadequate technical awareness, technical failures, inadequate technical manipulation, technical misuse, technical defects, technical immaturity, “black box”, technical uncertainty
7	Data risk	Hacking, compliant data behavior, biased data omissions, lack of hardware stability, data management gaps, poor data security, image recognition, voice recognition, smart home, data adequacy, false information
8	Privacy breach risk	Privacy breach due to data resource exploitation, privacy breach due to data management vulnerability, data breach, privacy breach, user knowledge, user consent
9	Managing risk	Deficiencies in the management of application subjects, inadequate risk management capabilities, lack of supervision, legal loopholes, poor risk-management capabilities, inadequate safety and security measures, inadequate liability mechanisms
10	Unemployment risk	Machines replacing humans, mass unemployment
11	Risk of ecological imbalance	High energy consumption in the development of AI, problem of asymmetry in biodiversity [31], disharmony between man and nature
12	Imbalance in the social order	Imbalance in the social order, social stratification, and solidification due to technological wealth disparity, imbalance in human–computer relations, social order, disruption of equity, uncontrolled ethical norms [32], social discrimination, digital divide, life safety, health
13	Autonomous controlled risk in human decision making	Substitute human decision making, machine emotions, AI entrusted with the ability to make decisions on human affairs, lack of ethical judgment on decision outcomes, participants and influencers of human decisions, changes in the rights of decision subjects
14	Risk governance	Educational reform, ethical norms, technical support, legal regulation, international cooperation [33]
15	Risk of unclear liability	Improper attribution of responsibility, unclear attribution of responsibility for safety, debate over the identification of rights and responsibilities of smart technologies, review and identification of attribution of responsibility, complex ethical subject matter [34]
16	Risk of inadequate decision-making mechanisms	Inadequate ethical norms and frameworks, inadequate ethical institution building
17	Decision judgment deficiency risk	Inadequate ethical judgment, poorly described algorithms for ethical implications, faulty instructions, complex algorithmic models, human-centered ethical decision-making frameworks
18	Decision making in team risk	Expert governance structures reveal limitations and shortcomings, illogical expert decision making structures, low levels of expert accountability
19	Consensus risk in decision making	Humans often disagree on solutions to real ethical dilemmas, no consensus, a crisis of confidence
20	Risk prevention	Enhance bottom-line thinking and risk awareness, strengthen the study and judgment of potential risks of AI development, carry out timely and systematic risk monitoring and assessment, establish an effective risk warning mechanism, improve the ability to control and dispose of ethical AI risks
21	Risk management	Awareness and culture of ethical risk management; establish a risk management department, risk identification, and assessment and handling; establish an ethical risk oversight department; development of internal policies and systems related to ethical risks; establish open lines of communication and consultation; establish a review mechanism for partners and risk reporting; focus on cultural factors and the significance of ethical risk management; governance with coordination
22	Ethical norms	Fairness, justice, harmony, security, accountability, traceability, reliability, control, right to control, good governance, social wellbeing

**Table 2:** Spindle code and main scope.

No	Main Scope	Initial Scope
1	Algorithm risk	Algorithmic discrimination risk; algorithmic security risk; algorithm interpretability risk; algorithmic decision-making risk; risk of algorithm abuse/misuse
2	Data risk	Data risk
3	Technology risk	Technical defect risk
4	Social risk	Unemployment risk; risk of ecological imbalance; imbalance in the social order; privacy breach risk
5	Management risk	Autonomously controlled risk in human decision making; risk of unclear liability; managing risk
6	Decision risk	Risk of inadequate decision-making mechanisms; decision judgment deficiency risk; decision making in team risk; consensus risk in decision making
7	Risk management	Risk prevention; risk management; risk governance; ethical norms

**Table 3:** Ethical risk variables and equations for AI decision making.

No	Variable	Type	Relationship Equation
1	Quality of decision-making teams	Constant	1
2	Employee accident risk rate	Constant	0.01
3	Decision-making mechanism	Constant	0.8 (assuming a 0.2 flaw in the decision-making mechanism)
4	Incident rate of a technical defect	Constant	0.2 (technical risk management can reduce most of the risk of technical defects)
5	Degree of algorithm interpretability	Auxiliary variable	“The incident rate of technical defect” × 0.5 + 0.2 (design discrimination in the algorithm itself + algorithmic black box issues)
6	Rate of algorithm abuse/misuse	Auxiliary variable	“Employee accident risk rate” + “Degree of algorithm interpretability”
7	Rate of algorithm security incidents	Auxiliary variable	“The rate of algorithm abuse/misuse” × 2 (algorithm abuse/misuse rate accelerates algorithm security incidents)
8	Decision consensus rate	Auxiliary variable	“Quality of decision-making teams” × 0.8 (assumes 80% consistency of decision making in absolute teams)
9	Risk of unclear liability for accidents	Auxiliary variable	“Decision consensus rate” × 0.2 (the higher the consensus rate of decision making, the lower the risk of liability accidents)
10	Algorithmic discrimination risk	Auxiliary variable	“Data risk rate” × 0.8 + 0.2 (much of the algorithmic discrimination comes from input data + algorithmic design discrimination)
11	Rate of a technical defect	Auxiliary variable	“The incident rate of technical defect” × 0.95 + “Employee accident risk rate” × 0.05 (a large part of this is due to technical defects and a small part to problems with the designers themselves)
12	Rate of algorithm risk	Auxiliary variable	“The rate of algorithm security incidents” + “The rate of technical defect” × 0.1 + “Algorithmic discrimination risk” × 0.1 (the algorithmic risk rate is in addition to the risk rate summarized by the current data. There are also risks that may be caused by future technologies and algorithms)
13	Rate of management risk	Auxiliary variable	“Risk of unclear liability for accidents” + “The rate of algorithm risk” + 0.2 (unclear responsibility for accidents and algorithmic risks can both contribute to management failures, coupled with the risks inherent in management)
14	Data leakage	Auxiliary variable	“The rate of technical defect” × 0.5 + 0.2
15	Data risk rate	Auxiliary variable	“Data leakage” × 2 + “Degree of social risk” (data breaches can accelerate data risk and are extremely risky for the data generated; the level of social risk also increases data risk)
16	Risk of imbalance in the social order	Auxiliary variable	“Privacy leakage” × 0.3 + 0.1 (privacy breaches can create social injustice by causing citizen panic and creating problems such as big data killings)
17	Privacy leakage	Auxiliary variable	“The rate of management risk” × 0.9 + 0.1 (privacy breaches are largely the result of mismanagement)
18	Risk of decision failure	Auxiliary variable	“The rate of management risk” × 0.9 − “Decision-making mechanism” (management failures can lead to decision failure and decision-making mechanisms can reduce the risk of poor decision making by at least half with a decision-making mechanism of 0.5)
19	Rate of decision risk	Auxiliary variable	“Risk of decision failure” × 0.9 + 0.1 (decision failure is a large part of the cause of decision risk)
20	Incidence of social risks	Rate variable	“The rate of decision risk” + “The risk of imbalance in the social order” + 0.1
21	Degree of social risk	Level variable	INTEG (“Incidence of social risks”, 1)

**Table 4:** Ethical risk governance variables and equations for AI decision making.

No	Variable	Type	Relationship Equation
1	Incident rate of a technical defect	Auxiliary variable	1-“Degree of risk management” + 0.1 (technology risk management, which reduces the risk of technical defects)
2	Data leakage	Auxiliary variable	“The rate of technical defect” − “Degree of risk management” (technical risk (including human) due to their data breach, but risk management will reduce the extent of the breach)
3	Risk prevention rate	Auxiliary variable	“Degree of social risk” × 0.9 + 0.1 (the higher the social risk, the higher the degree of the social risk equation, and the more ethical norms and management systems will strengthen the risk prevention rate)
4	Risk management rate	Rate variable	“Risk prevention rate” × 0.5 + 0.1
5	Degree of social risk	Level variable	INTEG (“Incidence of social risks” − “Risk management rate”, 1)
6	Degree in risk management	Level variable	INTEG (1-“Risk management rate”, 1)

## Data Availability

Data was obtained from [third party] and are available [from the References] with the permission of [third party].
